# Key Indicators and Social Acceptance for Bioenergy Production Potential as Part of the Green Economy Transition Process in Local Areas of Lapland

**DOI:** 10.3390/ijerph18020527

**Published:** 2021-01-10

**Authors:** Karetta Timonen, Anu Reinikainen, Sirpa Kurppa, Inkeri Riipi

**Affiliations:** Sustainability Science and Indicators Research Group/Bioeconomy and Environment Research Unit, Natural Resources Institute Finland (Luke), Latokartanonkaari 9 PL 2, 00791 Helsinki, Finland; anu.reinikainen@luke.fi (A.R.); sirpa.kurppa@gmail.com (S.K.); inkeri.riipi@luke.fi (I.R.)

**Keywords:** green economy, green growth, indicators, bioenergy, local level, ecosystem services, total sustainability, resilience, self-sufficiency

## Abstract

The aim of this article was to create key indicators for measuring the implementation potential of the green economy transition at a local level in a northernmost, natural biomass-rich environment. The case area to test the set of indicators was the village of Saija in Lapland. The work presented in this article is based on a communicative cooperative research and development project. The selection process for the appropriate indicators is based on a conceptual framework for developing local sustainability indicators and the thematic framework follows the key dimensions of the green economy (ecosystem resilience, resource efficiency and social equity). When selecting the local-level indicators, a strong emphasis was placed on the special characteristics of the local area and the availability and validity of the data. Layman villagers and data policy relevance (in this case green economy) were also taken into consideration. The key indicators developed as a result included: the increment of growing forest stock in relation to the drain on growing forest stock, the village population, the bioenergy consumption share, the utilization share of side streams, the bioenergy production potential, capital outflow, demographic dependency ratio, the ratio between employed and working age residents and the number of forest owners in relation to area households. The key indicators are targeted for use in supporting local decision-making and monitoring and assessing development activities and their effectiveness in the process of the green economy transition. The indicators measure the most critical factors for green economy transition in a local area and identify the most optimal development opportunities when moving towards green growth. In the measurement of the transition potential of the green economy, it was found that the case area’s imported fossil energy consumption could be substituted with self-sufficient bioenergy production utilizing the area’s own raw materials. There is extensive potential for the utilization of manure (an agricultural side stream) and forest resources (forestry side streams) at the local level, especially since forests account for 98% of Lapland’s land area. In support of the change from fossil-based energy consumption towards bioenergy production, plans for a biogas plant were examined for self-sufficient bioenergy production and this appeared to be the initial key process in the path of the green economy transformation in the case village of Saija.

## 1. Introduction 

Climate change requires ambitious political goals to reduce carbon emissions from all nations and within all sectors, especially those mentioned in the Intergovernmental Panel on Climate Change’s (IPCC) Fifth Assessment Report [1] (IPCC, 2014). Effective policies are required to achieve potential positive impacts on CO_2_ reduction (e.g., [2]). International agreements, e.g., the Paris Agreement [3] aim to reduce carbon emissions and reduce global warming. Furthermore, the EU’s Eighth Environment Action Programme, that guides the European Union’s environmental policies highlights climate-neutrality and resource efficiency [4]. According to the UN Environmental Programme (UNEP), “the green economy can be thought of as one which is low carbon, resource efficient and socially inclusive,” [5]. The green economy has been mainstreamed since the 2012 UN Conference on Sustainable Development in Rio de Janeiro (Rio + 20) [6] and is widely held in academia and policy making to be one of the key sustainability avenues [7,8]. Several global institutions have examined issues concerning the transition to the green economy [5,9,10,11]. 

There are already good examples of green economy innovations in the Nordic regions, for example local biofuel production from fish waste in Iceland, which has opened new business opportunities and contributed to job creation in the area, and forestry sector innovation in bioenergy in North Karelia in Finland, which has been stimulating regional wellbeing and resilience [12]. It has been stated by [12] that Nordic regions have great potential to build greener societies due to the considerable quantity of natural resources, good scientific knowledge, tradition of collaboration and being forerunners of cleantech and bioeconomy development.

Rural development is regarded as one of the most important drivers of the bioeconomy [13]. Rural raw materials form the regional basic resources for bioeconomic business activity. In the EU’s internal market, this issue is linked to the smart specialization strategy (3S), which emphasizes regional networking and partnerships across national boundaries and throughout the sectorial economy. According to [14], renewable energy production by utilizing already existing and presently unused resources can play an important role in vitalizing regional economies in rural areas, especially by substituting fossil energy and bringing the money spent on fossil energy back to be circulated in the regional economy.

The local level is a good level of governance for planning and making assessments [15]. The local level is different from the international level, considering cultural and historic traditions as well as physical closeness, which enables strong interaction and networks to evolve at the cultural and physical level [16].

Indicators can be applied by municipal administration for the self-assessment of internal processes, or for analysing overall performance within the local territory [17]. In order to achieve policy objectives and development, indicators at the local level are needed. The measurements made at this level can be further exploited at the provincial, regional, national and international levels. Indicators, and their measurements provide information for decision making at first at the local level, but they can be potentially used also for creating policies at other levels. Thus, the information produced by the indicators can be utilized in planning and decision-making processes [18].

Indicators are context dependent, and therefore it is essential to understand their development and their local context when developing an individual set of indicators [19,20]. Understanding the local-level context is important because local-level changes (e.g., deforestation or overgrown farmlands) may be significant as they affect an area’s scenic and recreational value but may not be relevant at the regional level. International and national level indicators are based on averages and local-level characteristics remain hidden under the more general-level information [21]. Utilizing average information may simplify the local situation and hide some essential aspects at the local level. In addition, some local-level indicators do not apply to other local areas due to the different characteristics of the areas [22,23]. Even though communities need to develop their own indicators, a shared structure for development is needed which enables comparison between communities taking into consideration the communities themselves, individual situations, and needs [19].

The aim of this article is to present key indicators for measuring the implementation potential and future effectiveness of processes in the green economy transition at a local level in a northern, naturally biomass-rich environment. In this study we refer to the local level as the village area. The case area was the village of Saija in Lapland. A key issue was to acknowledge the specific ecological, economic, and social characteristics of the localities in this region when formulating the set of indicators for the local green economy, but also to ensure a close interrelationship between the local and regional development processes. 

## 2. Theory: Green Economy Concept and Indicators 

There are variations in the definition and the concepts of the green economy used by different global institutions, research authors and nations [5,6,24,25,26,27]. For example, according to [26], moving towards a more sustainable future and a green economy means change and movement towards low carbon economic growth, ensuring the provision of functional ecosystem services and ecosystem wellbeing. According to [5], “The green economy can be thought of as one which is low carbon, resource efficient and socially inclusive”.

The concept of the green economy represents a combination of elements from both the circular economy and the bioeconomy (e.g., eco-efficiency and the renewability of resources). In addition, it endorses the underpinning role of a wide range of ecological processes, being more inclusive of some local aspects of the social dimension [8].

In this study, we applied three commonly accepted paradigms of the green economy: (1) to ensure ecosystem resilience, (2) to improve resource efficiency, and (3) to enhance social equity [27].

### 2.1. Ecosystem Resilience

Resilience was first defined by [28] as “a measure of the persistence of systems and of their ability to absorb change and disturbance and still maintain the same relationships between populations or state variables”. Gunderson [29] defined resilience as follows: “Resilience in ecological systems is the amount of disturbance that a system can absorb without changing stability domains.” In addition to these, resilience has numerous levels of meaning [30,31,32,33].

Ecosystems that lack resilience are vulnerable to disturbances that can lead to reductions in the supply of ecosystem services. The exploitation of ecosystem services should not exceed or endanger their long-term production [34]. The sustainable use of an ecosystem secures its ability to function, which in turn secures the continuum of productivity and the economy based on the ecosystem services. 

Ecosystem services are both immaterial (cultural, regulating, and supporting services) as well as material (provisioning services) provided by nature [35]. The concept of ecosystem services provides an applicable framework to elucidate the rate of the use of biomass resources for various purposes within the boundaries of global sustainability [36,37]. 

Ecosystems and human society are integrated [38], and ecosystem services are evaluated by people [35]. Economies cannot function without nature as our life support system. Unless we learn to properly value ecosystems with the finite resources which all life depends upon, we risk destroying them. Additionally, the carrying capacity should be considered, which is the maximum number of individuals for a given region that an area’s resources can sustain indefinitely without significantly depleting or degrading those resources [39].

### 2.2. Resource Efficiency

Resource efficiency has competing definitions (e.g., [40,41]. According to the European Commission [9], “resource efficiency means using the Earth’s limited resources in a sustainable manner while minimising impacts on the environment. It allows us to create more with less and to deliver greater value with less input.”

The cascading concept depicts the efficiency of resource use [42] (Keegan et al., 2013) prioritizing reuse of resources in a hierarchical order [43] (Sirkin and ten Houten, 1994). The industrial utilization and recycling of materials is required for higher added value products. Energy production is the least favoured option of material use after disposal as waste. Rytteri and Lukkarinen [44] have noted, however, that following strict cascading principles may problematically limit the use of bioenergy based on non-utilized and non-profitable materials elsewhere in decentralized energy plants of sparsely populated rural areas. 

### 2.3. Social Equity

According to Falk et al. [45] equity is connected to the idea of social justice, which refers to the satisfaction of people’s basic needs and equality in wealth distribution between different groups in society, as well as fair means (policies) to achieve these goals. A similar definition of equity is pointed out by Beder [46] and Jones [47] highlighting everybody’s right to a decent standard of living and fair distribution of wealth and income. The definition of sustainable development emphases equity between generations in terms of “development that meets the needs of the present without compromising the ability of future generations to meet their own needs” [48].

Fair access to resources and livelihoods is one of the aspects of equity [49], which directly or indirectly influence society’s finances and well-being. According to the Millennium Ecosystem Assessment [35], poor resource accessibility often leads to low human welfare even in natural-resource-rich areas. Further unjustified transitions in the equity structure of societies can lead to the unbalanced use of ecosystem services, e.g., those who have better access to capital have more opportunities to participate in capital- and technology-intensive resource exploitation.

### 2.4. Green Economy Indicators 

An indicator is a parameter, or a value derived from parameters, which points to, provides information about, or describes the state of a phenomenon/environment/area with a significance extending beyond that directly associated with the parameter value. Indicators represent the existing status of systems or situations and streamline communication by making information more coherent [50]. Indicators work as tools for communication, monitoring, and evaluation for comparison across time or space, or with other municipalities nationally or internationally, for follow-up of internal work, or as a way to identify problems and assess performance more widely within a local territory [17]. 

The number, quality, and scalability of green economy indicators are still new. Examples include the OECD [11] green economy indicators at the international level, and Seppälä et al. [26] and UNEP [51] for national or country-level indicators. All of these emphasize ecological and economic indicators but partly or totally lack a social dimension. 

There are no existing local and regional green economy indicators (GEIs) for northernmost, natural biomass-rich environments (e.g., Lapland areas covered by forest). However, there are some green economy indicators on the regional level in China, for instance. Wang et al. [52] utilize a driving force pressure–state–impact–response conceptual model (DPSIR) for a green economy development measurement index system. Even though the DPSIR model is widely acknowledged and utilized, e.g., by the European Environmental Agency (EEA) and Wu et al. [53], there are challenges with PSR models and their implementation in capturing economic and social dimensions [54]. Vukovic et al. [55] present regional system-level static and dynamic criteria and indicators for a Russian context. The methodological approach of the study combines data uncertainty and the present state and dynamics of the green economy. However, the indicators presented by Vukovic et al. [55] cannot be implemented as such at the local level as they focus more on the system-level dynamics of green economy development and most of them are lacking data at the local level. 

Sustainability including all sustainability dimensions (ecological, economic, and social) is seen as one definition for green economy thinking. There are some sustainability indicators published at the urban level [56,57,58,59,60,61,62], rural level [63,64,65], municipality level [15,17] and the community-based level [19,66,67].

## 3. Materials and Methods

### 3.1. Case Village Saija (Lapland, Finland) as Local Area Inside Lapland Region

The case area of this study is a village called Saija, which is located in North-Eastern Finland in the Salla municipality (67°05.63′N, 28°50.09′E). There are 136 inhabitants [68] and 60 households in the village. Agriculture and reindeer herding are the main industries. Saija’s forest resources are large (the forest area of the land register is about 16,000 ha), but agriculture forms the main everyday economic activity, alongside reindeer husbandry. The village reindeer owners can sell their reindeer meat to the village’s own processing company. The final culinary products are reindeer chips to be traded outside the village. The company and the product indicate the novel co-operation between the different village practitioners. A nationally important landscape site was determined in the village by the Lapland Environment Centre, and the villagers have actively participated in establishing and maintaining the site. Overall, the Saija village (http://www.saija.net/eindex.html) has fairly active internal collaboration between the different stakeholders, e.g., the village has an active village association which arranges events in the village (including the world’s smallest jazz festival “Saijazz” and publishes the village newspaper “Saijan Sanomat”). There are also an extensive number of project activities, and a strong interest from key local stakeholders in building businesses, especially in the restaurant sector. Thus, the tradition of cooperation and collaboration is strong in the village. 

### 3.2. Lapland as a Region in Europe

Finland is Europe’s most forested country in terms of land area as 78% of Finland’s total area is forest. In Finnish Lapland, the amount of forestry land is 9.1 million hectares, which is 98% of Lapland’s land area. The state of Finland is the largest forest owner and there are 25,000 private forest owners in Lapland. Nature conservation areas and statutory wilderness areas cover one third of the Lapland land area: this includes 1 million hectares of forest, which means that more than 20% of the productive forests in Lapland are strictly protected [69,70]. Forestry and agriculture and the processing industry based on these can play a key role in providing bio-based substitutes for non-renewables [71,72].

### 3.3. Indicator Framework and Selection Process

There are six different types of frameworks for selecting indicators [73]. In this study, the fundamental framework for starting the indicator work is the domain-based approach [54,73]. Originally Maclaren [74] gave an overview of indicator frameworks, which was further modified by Page [73]. Six different types of domains have been identified: domain-based, goal-based, sectoral, issue-based, causal and hybrid. Our sustainability research approach was domain based, but from the start we were aware of the trade-offs at the interfaces between different dimensions of sustainability. Our domain-based approach was analogical to the domain model for energy of Nathan & Reddy [54] for their urban context. The strong national and regional policy regarding the green economy introduced the goal orientation. A sectoral framework was something that we systematically tried to avoid, because that would have led to simplified economics of scale and as such would have killed the inclusive idea of the villagers. Our issue-based approach was secondary. Causality was the key issue in our holistic village approach, but our framework can be regarded as hybrid. We wanted to invite the villagers along with regional administrators to work at a meta-level instead of directly selecting a set of indicators. We searched for multidimensional information and built up connections to a few key indicators. We aimed at indicators that would be conceived from multiple angles. 

This leads to a process of establishing indicators which is causal [75] and the driving force–state–response (DSR) model was applied as the basis for the key indicator selection. The pressure–state–response (PSR), the driving force–state–response (DSR) and the extended version driving force pressure–state–impact–response (DPSIR) are frameworks (conceptual models) for indicator selection that consider the causal chain (cause and effect) of the indicators [75]. In the driving force–state–response (DSR) model, “driving forces” refer to forces affecting the environment, creating changes in the “state” of the environment and with a societal “reaction” to these changes [76]. The DSR model was applied as the basis for the key indicator selection. The green economy was a tool to change the situation of the village and increasing renewable energy production was the most important way to achieve this in the context. The need (driving force) to utilize renewable natural resources in a more efficient way (measured by resource efficiency indicators) affects the state of the village environment, measured using resilience indicators which reveal the societal effects on the village demographics (response). These are measured using social equity indicators. 

**Figure 1:**
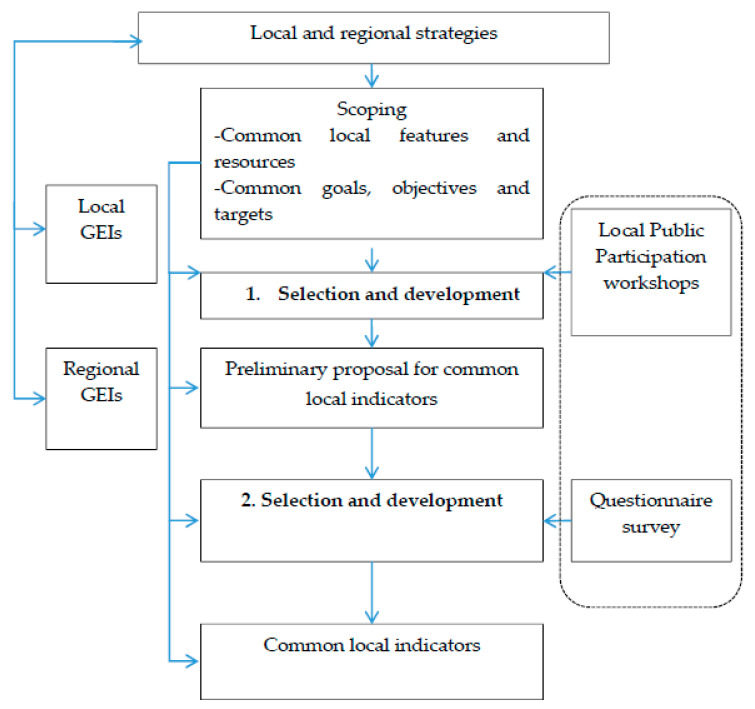
Conceptual framework for developing local indicators [77] (applied from the original study [15]).

Finally, the selection process concludes with operative steps applying the sustainability indicators selection process framework. In this study, we applied a conceptual framework for the development process of indicators at the local level [15] (Figure 1). The operative process stages (Figure 1) were applied to develop green economy key indicators for northern, biomass-rich village environments.

As a first step (in Figure 1), the developed indicators must use strategies on a local and regional scale. The Lapland region was one of the frontrunners in adapting smart specialization strategies (3S), which are innovation policy concepts aiming to increase employment and growth by enabling regions to identify and develop their own competitive advantages in Europe [78]. Lapland has been determined as one of the European Commission’s 3S model regions and as a target for cluster development [79]. In Lapland, the approach, for example, focuses on developing novel businesses in decentralized renewable energy production to reduce the outflow of capital from rural Lapland [80]. The Lapland Agreement is a provincial program and a statutory strategy that will guide the development of the province over the next four years. It describes the key themes and strategic choices that various actors in the province will focus on in the coming years. It highlights aspects such as the utilization of local natural resources and resource-efficiency. At the local village level there is no strategy available. The regional strategy should be reflected by the local approaches. The transposal of the higher-order regional strategy to local level was performed by a Back-casting process [81]. In Back-casting, the regional goals were, in a participatory process, formulated as the final objective of the villagers. Transformation from being users of outsourced fossil-based energy services to becoming self-sufficient providers of renewable energy was targeted. This would be a long-term stepwise and holistic process for a village. 

As a basis for indicator development work, there is a need to map the already existing local and regional green economy indicators (GEIs). These were not to be found in the specific case area in Lapland. However, we applied some of the already published green economy indicators (see Section 2.4), for example, some national-level green economy key indicators [26], e.g., the increment of growing stock in relation to the drain on growing stock, and bioenergy consumption share in relation to fossil energy. In addition, we utilized commonly used socio-demographic indicators. 

The scoping for local features and natural resources was carried out firstly by experts at the ProAgria Lappi Rural Advisory Services and the Finnish Forest Centre, due to the expertise needed to specify the key indicators, and to compile and utilise the data [20]. The prevailing local structure was mapped for the assessment of local potential for green economy transition. The migration of local residents was observed, and many services (e.g., health care, postal services) were being reduced and moved further away from the local area. This was the status of the socio-demographic and socio-economic factors that were to be critically examined as part of this research project. When the data was collected on the renewable natural resources of the local area, it appeared that there was a large reservoir of low-profit use of resources, in addition to unutilized forestry side streams and manure. A large amount of unrenewable energy consumption was totally based on imports. 

The preliminary goals, objectives, and targets were defined by experts at the ProAgria Lappi Rural Advisory Services focusing on the development process of energy systems with potential for self-sufficient bioenergy production. Previously unutilized forestry side streams and manure were determined as the main source for self-sufficient and sustainable substitution for the imported fossil energy. Capital for imported fossil energy was found to be critical. The expert study noted that capital should not flow out of the area but bring economic growth into the area. Potential was seen to generate jobs in decentralized energy production and to promote population growth. This was seen as the first step on the way to creating a base for a sustainable infrastructure, to mobilize resources in the area, and to build a base to advance local livelihood vitality. In the long run, this would be a foundation for green productivity and green growth. In the post-renewable energy era, this should open the way to totally emission-free energy production (e.g., wind and solar) when biomasses can be reutilized for high-profit and long enduring products, according to the cascading principle (Chapter 3).

Local-level green economy indicators were further elaborated in cooperation with local residents in workshops. Opportunities to participate in village meetings were provided and 13% of residents attended. The attendants were important decision-makers in the village who are key persons in building trust with other residents. The importance of local participation has been acknowledged in several sustainability-indicator development studies [19,20,66,82,83,84,85].Villager involvement in the conceptualization of the indicators is crucial, to include their personal views, individual values, concerns, and mutually prioritized goals [19,86]. The indicator research approach was integrated with the experiential knowledge of local participants (including indigenous people). The Natural Step approach [81,87,88,89] was applied and implemented in the village meeting to create, together with the residents, a more defined and prioritized vision of the local area. Participants were divided in groups and assisted with a facilitator from the project group. The aim was to clarify the current situation of the village, the desired vision for the future, as well as possible obstacles and means to achieve the vision. Information about the local features and natural resources as well as preliminary goals, objectives, and targets defined by experts at the ProAgria Lappi Rural Advisory Services presented above was used to help local people discover and realize the potential of their villages. The aim was to highlight the different possibilities of the village bioeconomy through discussions and presentations, and at the same time to analyze the starting points of the village’s operating environment and the inhabitants’ attitude to change. The events were dialogical, aiming to motivate the villagers themselves in the development work and in concrete brainstorming about new businesses, living in the village and visions for the future of the village. The village meeting revealed that the villagers are concerned about the future if young people move away due to a lack of jobs and they are willing to act together to change the future development of the village. Young people revealed their willingness to live and work in the village and start their own businesses. For example, they showed interest in novel business opportunities which could occur in the renewable energy and food sectors. The desired vision of the future was focused on a decentralised biogas plant for self-sufficient bioenergy production, replacing the present energy demand being met by fossil fuels. With the biogas plant, energy self-sufficiency is created, which eliminates capital flight. Biogas plant utilizes waste (unutilized side streams), increases employment in the area, and prevent the population from continuing to decline and move out. 

*The first stage of selecting and developing the* indicators was to bring together the results from the previous steps and aim to present the first set of indicators measuring the potential for biogas production in the area (e.g., sustainable raw material base) and its targets (e.g., self-sufficient bioenergy production potential, potential for eliminating capital flight). 

The preliminary proposal, in other words, the first set of locally prioritised green economy indicators, was presented to the project steering group, which consisted of experts in the bioenergy field (from the Ministry of Forestry and Agriculture, and the Ministry of Economic Affairs and Employment), experts in sustainability indicators (from the Ministry of the Environment), and experts in regional and rural development (from the Nordregio and Regional Council of Lapland). After this, the indicators were discussed further within a project group of sustainability research scientists (Natural Resources Institute Finland, Luke), which went through the common indicator criteria to confirm their acceptability [90], reliability [91], feasibility [22], measurability [22,23,92] and accountability [93,94]. Indicators should be widely accepted [90]. The “acceptability” of local green economy indicators is based on the literature (studies conducted) and national and international reports [11,26] as well as cooperation with experts and local residents. The proposal for the selected green economy indicators was discussed in the project steering group (expert panel). The acceptability, trust, legitimacy, and support of the development plans were tested and evaluated using a questionnaire for local residents. The criterion of “measurability” was met because the values of the indicator could be measured and the measurement repeated, i.e., the measurement would give similar results under similar conditions over time [22,23,92]. Data availability is essential to select the most appropriate indicators to measure green economy data now and in the future. It is necessary to identify indicators that can be used to collect monitoring data using new methods. The availability criteria were already assessed during the collection work and only those indicators were considered in the selection from which data could be collected now and in the future at the village level and at the level of Lapland province. Statistics were often available at community level and local data had to be collected through interviews. According to Dubnic & Frederickson [94] “Being accountable” means transparency, responsibility for one’s actions, and subjecting oneself to control and guidance. Hall & Ferris [93] state that accountability “perceived expectation that one’s decisions or actions will be evaluated by a salient audience and that rewards or sanctions are believed to be contingent on this expected evaluation”. To meet the criteria of accountability the data collection was done by experts and local residents. National statistics were the main source of the statistical data and can be stated to be transparent, providing good grounds for the data gathering and calculation in terms of accountability. According to Puolimatka [91], “reliability” criteria refer to the ability to provide reliable information about the field to which research seeks answers. To ensure reliability, experts collected and analyzed the data and workshops were held with local residents to obtain best practice and reliable information. The “feasibility” of the indicators was ensured by evaluating the data collection method and cost-effectiveness, and defining the data collector, issues highlighted by Miller & Twining-Ward [22].

In the second stage of selecting and developing the indicators, the local-level green economy indicators were tested and evaluated further with a questionnaire survey analysing the acceptability, trust and support for the indicator values and development plans (biogas plant in the area) by local residents. The survey was conducted in all the 60 households of Saija of which 41 were interviewed and the rest either rejected the interview or were not reached. The respondents represented 75% men mostly over 40 years of age (within the village, 57% of the residents are men and 77% of the residents are over 40 years old [68]. About 40% of the respondents were workers and entrepreneurs and 8% were unemployed (about 38% of the village residents were workers, 4% were unemployed and 45% were retired [68]). Nearly half were retired; some of them had moved to the village at the beginning of their retirement. Half of the respondents were involved in the village association and almost all had lived in Saija their whole lifetime. The results indicated approval by the residents since 68% of the residents answered the questionnaire and 90% of the questionnaire respondents believed that the energy calculations presented by the project were credible and approved the development plans. Almost all respondents (95%) said they were aware of village development plans (e.g., biogas plant). Most respondents considered the plans clear and understandable, 68% considered the energy calculations credible, and 25% could not say anything about credibility (only 7% were not convinced about the calculations). Over half of the respondents (63%) considered profitably calculations realistic, 28% could not say anything and 10 % considered them unrealistic. Two thirds of the respondents were convinced that the biogas plant would be built in the village eventually and the majority of the respondents (90%) were sure that the plant would benefit all the village residents. Further, 75% of the respondents were content with the planning process e.g., it had been unanimous, equal, and sharing of information had been open and sufficient. Three-quarters (80%) of the respondents were sure that the biogas plant could provide jobs and new residents in the village. In addition, 90% of the respondents trusted the development plans’ ability to achieve the village’s vision. To conclude, the respondents were interested in the development project regarding the biogas plant. The open comments about the project regarded the practical implementation and actual costs and investment needs of the biogas plan.

## 4. Results and Discussion

Based on scoping the local features and resources and exploring the common participatory goals, objectives, and targets, indicators for the defined and measured transition potential were developed. These were to support the change from fossil energy consumption towards bioenergy production, based on planning a biogas plant in the area for self-sufficient bioenergy production and consumption. Every key indicator meets one of the three green economy dimensions (ecosystem resilience, resource efficiency, or societal equity). The indicators also gained social acceptance. The local level key indicators (Table 1) reflect the potential in moving towards the green economy through bioenergy production. 

### 4.1. Ecological Resilience

The first key indicator presented in Table 1 “The Increment of growing stock in relation to the drain on growing stock” describes the sustainability of the forest stock. It focuses on maintaining the productivity of the ecosystem services (provisioning services e.g., wood and berries) as well as the sustainable raw material base for forestry side stream production and utilization as raw materials in bioenergy production. In addition to productivity services, it focuses on maintaining cultural services, e.g., recreational services, regulating and supporting ecosystem services e.g., photosynthesis and carbon circulation), thus ensuring functional ecosystems. The forest represents a key resource in terms of ecological resilience in the targeted northern context of this study. Saia’s forest resources are large and the annual drain (annual depletion) of the growing of stock of forests is no larger than the increment of the growing stock. Additionally, from the regional perspective, in Lapland, more than 20% of the productive forests are strictly protected [69,70]. Next to this simplified key indicator, it should be noticed that estimating the usage of forest resources still lacks holistic ways to incorporate ecological and social sustainability into the models [96]. 

The ecosystem services have impacts on socio-demographic factors (e.g., the “village population”, Table 1) in the area, which have implications for local people’s welfare [35]. Peripheral regions in Lapland, where young people are moving away and growth in the working aged population is needed, migration is a current and potentially growing risk in the context of achieving sustainability goals [97]. In the case village of Saija, the population has been decreasing since the 1990s when the inhabitants numbered slightly below 200. At this moment, the main reason for the population decrease is the lack of jobs. It is hoped that future bioenergy production will attract more jobs and population to the area and therefore also improve the resilience of the local society. The village population is also a factor contributing to environmental carrying capacity (the amount of people that the area’s resources can sustainably sustain). In many parts of the world there are too many people fighting for scarce resources and against the limits of the ecosystems come against. Poverty and the daily struggle to survive disable concern over depletion of resources or expansion of pollution. However, population growth is linked to society’s resilience in sparsely populated Nordic areas. The immersed renewable natural resources require some local volume (people) to be utilized and people’s faith in living possibilities in the area, otherwise the utilization potential drifts outside the area to other national or international actors. Measuring the optimal number of people in the area and environmental capacity is challenging here because one needs to take into consideration preserving not only physical but also social and cultural viability [39] and there is still a lack of suitable measures and data to evaluate these aspects.

Minimizing the environmental impacts of energy products enhances the environment’s resilience. The “bioenergy consumption share” (Table 1) in the area has a high potential to grow, replace non-renewable energy and fulfil the area’s total energy consumption needs. This aims at carbon neutrality as one of the green economy goals. In this study, we were not measuring environmental impacts (e.g., life cycle assessments (LCAs) which assess the environmental impacts) of biogas production as key indicators, since LCIA methods use temporal and non-spatial borders, from cradle-to-grave, for this sort of measurement, without spatial borders as local level indicators. The same situation exists for other life cycle methodologies related to the evaluation of economic (Life Cycle Costing, LCC) and social impacts (Social Life Cycle-Costing, S-LCA).

### 4.2. Resource Efficiency 

Unutilized side streams indicate aspects of the circular economy and resource efficiency in the area. Next to forestry side streams and manure, there are a few other potential unutilized waste streams to be found in the case area, e.g., household waste, reindeer slaughter waste, and surplus grass. However, in this study, for the key indicator “the utilization share of side streams” in Table 1, we assessed the unutilized shares of forestry side streams and manure in relation to the already utilized shares since they were the most profound in the case area of Saija. Considering unutilized forestry side streams and manure as waste, these streams should be first prevented, then reused, recycled, and finally utilized in producing energy, according to cascading use [98]. In this study, when taking into account the case area characteristics, it was assumed that following a strict EU waste framework would narrow the possibilities for biomass utilization in the Lapland context, as also mentioned in the study by Rytteri and Lukkarinen [44]. The decentralized organization of the bioeconomy could promote benefits in rural areas as mentioned also in the study by Pfau et al. [99]. Enhancing the utilization of side streams including smaller production systems would increase economic well-being locally [100,101,102]. There is a risk of residents moving elsewhere if possibilities to earn money from the present resource base are not quickly enough improved, in the area.

“The bioenergy production potential” (Table 1) locally is assessed from the share of burning wood for energy and other forestry side streams. Energy calculations were carried out by experts at the ProAgria Lappi Rural Advisory Services. Through the transition process to the green economy, an area such as the village of Saija has the potential to meet its total energy demand for fossil energy by producing energy from its own manure and forestry side streams. The production potential is greater than the demand for fossil energy and therefore leaves the potential for an energy production surplus to be utilized and sold somewhere else. However, this potential was not assessed since this study’s key indicators concentrated on meeting the self-sufficiency targets. 

Utilizing forestry side streams as raw materials for bioenergy works as an intermediate step towards a sustainable base infrastructure for green economy business-making locally. In the future, even better renewable energy sources such as wind and solar power will be utilized which will enable biomass utilization for more highly prized uses.

“The Capital outflow” in Table 1 reflects the monetary value that is lost outside the area by consuming and importing products that can be substituted with production from the area itself (for example, utilizing side streams for bioenergy production). One of the emerging challenges found in this study is that there are yet no market prices for forestry side streams and renewable energy products, in the area. Decentralized renewable energy production is seen to cut off the outflow of capital from rural Lapland. At this moment in Saija, the capital of private individuals and corporations is escaping from the village because of the consumption of unrenewable energy produced outside the area. This capital could be used to maintain the village area if local resources were utilized for bioenergy production. In this case, capital outflow is a way to measure the value of self-sufficient renewable energy and its raw material (side stream) production in the area. 

### 4.3. Social Equity

“The demographic dependency ratio” as a key indicator describes the demographic state determining local vitality [103]. People who are working face a great burden in backing up the ageing population and therefore the number of people of working age must grow for sustainable demographic development in the area and for better social equity and wellbeing. There are young people and potential returnees, but at this moment young people are moving out of the area to the cities due to the lack of jobs. Job opportunities are a fundamental basis for younger people to remain in the area. 

The decentralized organization of the economy has the potential to increase well-being in the countryside [99] and foster social benefits through local employment [102]. The “employment rate” means the number of employed residents in relation to all working age residents and it has been decreasing in the area during past years. However, the green economy transition process (bioenergy plant) in the near future may attract more jobs and improve the employment rate in the area. Challenges in achieving this include the difficulty of attracting a qualified labour force, especially to smaller and rural regions [104,105]. Education, new knowledge, apprenticeships, improved competence in the green economy (e.g., in bioenergy production) are needed. Lifelong learning is necessary, and the village residents have good opportunities for this as most of them have a professional degree. Next to bioenergy production, it should be noted also that tourism as an example of a cultural ecosystem service has become an important economic sector now providing more job opportunities than the forest sector. Lapland has abundant natural assets and nature is the cornerstone upon which the rapidly growing (circa 10% during previous years) tourism sector depends [106]. However, tourism may disturb the welfare of ecosystems if it is followed by a disposable culture. The growing popularity of ecotourism is challenging the earlier business models and ecosystem wellbeing needs to be considered as a provider of tourism in the long run.

Ownership represents who has control over the resource utilization, and equity to gaining value from ecosystem services. The raw material base for the green economy transition process reflects social equity through ownership and, in this way, access to resources. The more “Forest owners in relation to area households” that there are, the more social equity will manifest. Although the State of Finland owns a large proportion of the forest area in Saija, every one of the 60 residents are private “forest owners” and own at least a small share of forest land and its wood resources (sharing the utilized side streams). In terms of resource (forest) ownership, the situation in Lapland is complex as there are large state own forests and areas where indigenous people have the right to practice reindeer herding without ownership. Additionally, all village residents have opportunities to be partners in the biogas plant company as local ownership leads to better acceptance in planning local level renewable energy production [107,108].

### 4.4. Uncertainty and Sensitivity of the Indicators 

The presented group of key indicators have been designed based on the scoping of the village economic, environmental, and human resources and a participatory governing process for defining the strategic green economy approach with specific targets for the village. The key desire is a positive development of the village, which means effectively utilising the potential of green economy. In the potential for success of the green economy, bioenergy production potential, utilization share of side streams and prompt minimising of capital outflow are the leading indicators. First indications of change should be seen through these indicators. Change in the bioenergy consumption share should come consequentially. Ratio between employed and all working age residents and demographic dependency change more gradually. Changes in village population might be quite unexpected as movement by a few families in or out in such a low village population makes a big change. Losing just a few key villagers or families might ruin the potential for the expected transformation. This happens if involvement in the basic strategic approach weakens. The increment of growing forest stock in relation to the drain on growing stock, and forest ownership are expected to be most stable.

During this process of indicator development, several mutual assumptions have been made that affect the certainty and sensitivity of the indicators. During the targeted transformation to a green economy, the basic economic and social structure of the village is expected to follow the change without any radical disruptions. Farming and reindeer herding are expected to continue. The reindeer chip processing company is expected to continue its activity. Ecotourism is expected to gradually develop, but its impact in present indicators is minor. The distribution of any pandemic to human, animal or plant populations was not foreseen. Climate change continues inevitably and slowly. In this part of the world, the impact is expected to remain positive in terms of forest growth if correctly managed [109]. In this region of Finland, air is exceptionally clean, and no excessive distribution of harmful air pollution in the form of chemical, aerosol, black carbon etc. were considered in indicators of the development process. Energy production technology was expected to manage the avoidance of these polluters in the village and around.

The leading green economy indicators are, of course, sensitive to national and regional policy interventions and price relations between renewable and fossil-based energy, and remarkably sensitive to national policy in supporting or not supporting investments in renewable energy production facilities. The present governance in Finland is strongly involved in the Green Economy, but questions remain as to how allocation of funding will be directed between rural and urban communities. Exceptional sensitivity to all resource efficiency indicators is also caused by the closeness of the Russian border. Russian fossil-based energy is cheap and there is a strong temptation in some of the villagers to fill up with car fuel from the Russian side. Trade connections over the EU outer border might have a radical impact on this threat. Changes in mutual trust among the villagers in the developed strategy for green economy is something for which we did not manage to build up an indicator.

The presented key indicators are transferable to other localities in the northmost biomass rich region, in which the requirement for similarity of local features and involvement in the green economy strategy and the commitment of national and regional policy approach to the green economy would be sufficiently fulfilled.

### 4.5. Criteria for Choosing Indicators

One issue that was particularly challenging in the development of the key indicators was the accessibility and applicability of the data used. This issue was raised also by the European Commission’s Joint Research Centre, JRC [110] in the context especially of developing composite indicators. Indicators need to be policy relevant to become motivating. In our case, the policy linkage was to the green economy. Additionally, the information gathered for the indicators needs to be easily understandable and simple enough for the target audience. This was ensured in our case by the participatory approach. For validity, the indicators need to provide a true reflection of the facts, and the data collected must use scientifically defensible measurement techniques. Furthermore, the indicators need to be verifiable and reproducible, and it must be ensured that the data is credible for both experts (regional administration) and laypeople (villagers). Eight of the nine indicators that we selected are widely used in various contexts (e.g. [26]). The measurements follow scientific principles, and in the participatory process these appeared credible to the villagers. Capital outflow as an indicator is a less common indicator, but the dynamics between different forms of capital have been scientifically well elaborated in the context of rural poverty traps and the alleviation of poverty through resilience and transformation [111,112]. A relevant time series of the data needs to be available and must be capable of reflecting the trends of the indicator over time. This needs to be ensured in the assessment process. We tried to enhance this by building good connections with the regional administration (regional council) responsible for the databases of interest. The regional council is responsible for the quality of the data and for the cost of the monitoring process. At the local village level, indicators must detect any small spatially based change in the system. This is the key message of this paper to the regional administration. After the long development process, we cannot see any reason to question the reliability of the indicators. When the target villagers are part of the process, one can expect them to have high motivation to ensure that collected data is correct.

## 5. Conclusions

The focus of the article was to reveal key indicators for measuring the execution potential (and the effectiveness) of the transition process to the green economy at the local level in Finnish Lapland. The study sought information with indicators on aspects linked to ecosystem resilience, resource efficiency, and social equity.

Social acceptance of the developed indicators was aided by the project steering group, and trust in the energy calculations and development plans was demonstrated by most local residents. The tracking of sustainable use indicators is best realized by simultaneously developing systemic data collection systems, which can be used as databanks for raw material reserves, as well as data collection platforms for the relevant actors of the area. When concerning scalability, measures for decentralized systems must hierarchically gather data from the corporate, local, regional, and the national level. Indicator development work concerning the potential and the role of the local network in the transition process towards the green economy must be a further research topic. The indicator values can later be utilized to support the preparation of development policies for the village towards the green economy. Reproducibility and availability of data collection locally and throughout the Lapland Province will be important in the future. 

A significant amount of energy produced from fossil fuel is purchased from outside the area though there is a significant potential for self-sufficient bioenergy production based on sustainable resource utilization. Additionally, migration away from the area is a current risk and the employment rate is decreasing in the region. Indicator measurement results revealed that there are enormous amounts of unutilized raw materials (specifically forestry side flows and manure) for bioenergy production which could meet the entire local demand for energy and could substitute fossil energy consumption. The transition process to the green economy (bioenergy plant) in the near future will attract more jobs and lead to growing employment in the area.

Reusing resources (forestry side streams and manure) as raw materials for bioenergy would be an intermediate step and a first step towards creating a sustainable base in the local infrastructure for the green economy. After this other renewable energy production methods such as wind power can be brought into use, and biomass can be reused in more valuable products, in line with sustainable utilization, meeting the cascading principles of prioritizing high added-value products over energy production. In addition to energy production, there is a need to connect the energy sector to food production in the future. More information is needed concerning regulation and support for ecosystem services. In turn, these services determine the dynamics and regeneration ability of provisioning and cultural ecosystem services.

## Figures and Tables

**Table 1:** Developed green economy key indicators in local area.

	Local Indicator	Description	Unit of Measure	Baseline Value	Green Economy Vision in the Year 2030
**ECOSYSTEM RESILIENCE**	The increment of growing stock in relation to drain on growing stock.	Yearly growth of the amount of the natural resource (e.g., forest) is equal or greater than the yearly use of the resource.	ratio	35,000 m^3^/35,000 m^3^ = 1.0 The total growth of forest in the Saija area is about 35,000 m^3^ per year and is the same as the drain per year [70].	35,000 m^3^/(> 35,000 m^3^) > 1.0 The target value will be one or more, in other words the forest stock of Saija village is growing more than the drain on the growing stock.
	Village population	When the number grows it represents an increase in the people living in the village area.	inhabitants	136 inhabitants The population has been decreasing and now there were 136 inhabitants in the village of Saija in 2017 [68].	>136 inhabitants The population will have grown and there will be more than 136 inhabitants in the village.
	Bioenergy consumption share	The bioenergy consumption share needs to grow and compensate for unrenewable energy consumption.	%	1613 MWh/6565 MWh × 100% = 25% The share of bioenergy consumption share of the area in relation to total energy consumption in the area is 25%.	6565 MWh/6565 MWh × 100% = 100% The share of bioenergy consumption in relation to the total energy consumption in the area will be 100%.
**RESOURCE EFFICIENCY**	The utilization share of side streams	Increasing utilization share of produced side streams in the area must grow.	%	1176 m^3^/8750 m^3^ × 100% = 13% The utilization share of forestry side streams is 13%. At this moment 1176 m^3^ of forestry side streams are utilized as heat energy. The total potential of forestry side streams is 8750 m^3^ which is 25% of total growth of forest trees (35 000 m^3^) [95]	8750 m^3^/8750 m^3^ × 100% = 100% The utilization share of forestry side streams will be 100%
			%	0 m^3^/ 4784 m^3^ × 100% = 0% The total potential of unutilized manure is 4784 m^3^/a. Manure has not been utilized at all and the share is therefore 0%.	4784 m^3^/ 4784 m^3^ ×100% = 100% The utilization share of manure will be 100%
	Bioenergy production potential	Bioenergy production potential through utilizing total side-stream potential.	%	1613 MWh/14,903 MWh × 100% = 11% The bioenergy production share of the total bioenergy production potential is 11%.	14,903 MWh/14,903 MWh × 100% = 100% The total potential for energy production is met by utilizing wood and other forestry side streams will be 12,000 MWh, and from manure 2903 MWh altogether 14 903 MWh per year.
	Capital outflow	The amount of money escaping from the area due to imported unrenewable energy must decrease.	€	688,236 € per year The value of imported fossil energy (4952 MWh) that is possible to be replaced with the area’s own bioenergy production (14,903 MWh) from forestry side streams and manure.	0 € per year The consumption of imported fossil energy and the monetary value escaping locally because of consumption of unrenewable energy will be zero.
**SOCIALEQUITY**	Demographic dependency ratio	The number of people of non-working age (0–14 and over 65 years) in relation to working age people (15–64 years).	%	63 residents (aged 0–14 and over the age of 65)/73 residents (working aged 15–64) × 100% = 86% In the Salla municipality area the trend is that the number of working aged residents is decreasing and the number of people over 65 years is increasing. It is estimated than by 2031 the ratio will be 1:4. e.g., almost 1.4 non-working aged per one working age person [68].	73 residents (aged 0–14 and over the age of 65)/73 residents (working aged 15–64) × 100% = 100% The demographic dependency ratio should not be weakening in the case area and should be close to value 1
	Employment rate	Generated employment in the local area reflected to the total number of the workforce (working aged 15–64).	%	20 employed/73 working aged × 100% = 27% There are 20 jobs mainly in the agricultural sector providing potential resources for bioenergy production.	23 employed/73 working aged × 100% = 32% It is assumed that a local bioenergy facility would on its own generate three jobs in the case area and will increase the employment of 5%.
	Forest owners in relation to households	The bigger the ratio the wider ownership of the bioenergy resources.	%	60 private resource owners /60 households × 100% = 100% All 60 residents are private forest owners in the area sharing the resources.	60 private resource owners/60 households × 100% = 100%The ratio will remain close to 1 reflecting possibilities for equal decision-making processes related to resource management in the area.
